# Association of significantly elevated plasma levels of NGAL and IGFBP4 in patients with diabetic nephropathy

**DOI:** 10.1186/s12882-022-02692-z

**Published:** 2022-02-11

**Authors:** Hamad Ali, Mohamed Abu-Farha, Eman Alshawaf, Sriraman Devarajan, Yousif Bahbahani, Irina Al-Khairi, Preethi Cherian, Zahra Alsairafi, Vidya Vijayan, Fahd Al-Mulla, Abdulnabi Al Attar, Jehad Abubaker

**Affiliations:** 1 Department of Medical Laboratory Sciences, Faculty of Allied Health Sciences, Health Sciences Center, Kuwait University, Kuwait City, Kuwait; 2 Department of Genetics and Bioinformatics, Dasman Diabetes Institute (DDI), Dasman, Kuwait; 3 Department of Biochemistry and Molecular Biology, Dasman Diabetes Institute (DDI), Dasman, Kuwait; 4 National Dasman Diabetes Biobank, Dasman Diabetes Institute (DDI), Dasman, Kuwait; 5 Medical Division, Dasman Diabetes Institute (DDI), Dasman, Kuwait; 6 Department of Pharmacy Practice, Faculty of Pharmacy, Health Sciences Center, Kuwait University, Kuwait City, Kuwait; 7 Diabetology Unit, Amiri Hospital, Ministry of Health, Kuwait City, Kuwait

**Keywords:** DN, NGAL, IGFBP-4, IGFBP, diabetes

## Abstract

**Background:**

Diabetic nephropathy (DN) is a type of progressive kidney disease affecting approximately 40% of patients with diabetes. Current DN diagnostic criteria predominantly rely on albuminuria and serum creatinine (sCr) levels. However, the specificity and reliability of both markers are limited. Hence, reliable biomarkers are required for early diagnosis to effectively manage DN progression.

**Methods:**

In this study, a cohort of 159 individuals were clinically evaluated and the plasma levels of NGAL, IGFBP-1, IGFBP-3, and IGFBP-4 were determined using Multiplexing Assays. Additionally, the association between the plasma levels of NGAL, IGFBP-1, IGFBP-3, and IGFBP-4 in patients with DN were compared to those in patients with T2D without kidney disease and control participants.

**Results:**

Circulating level of NGAL were significantly higher in people with DN compared to people with T2D and non-diabetic groups (92.76 ± 7.5, 57.22 ± 8.7, and 52.47 ± 2.9 mg/L, respectively; *p* <  0.0001). IGFBP-4 showed a similar pattern, where it was highest in people with DN (795.61 ng/ml ±130.7) compared to T2D and non-diabetic people (374.56 ng/ml ±86.8, 273.06 ng/ml ±27.8 respectively, ANOVA *p* <  0.01). The data from this study shows a significant positive correlation between NGAL and IGFBP-4 in people with DN (*ρ* = .620, *p* <  0.005). IGFBP-4 also correlated positively with creatinine level and negatively with eGFR, in people with DN supporting its involvement in DN.

**Conclusion:**

The data from this study shows a parallel increase in the plasma levels of NGAL and IGFBP-4 in DN. This highlights the potential to use these markers for early diagnosis of DN.

**Supplementary Information:**

The online version contains supplementary material available at 10.1186/s12882-022-02692-z.

## Introduction

Diabetic nephropathy (DN) is a kidney-related complication that affects approximately 40% of patients with type 1 and type 2 diabetes mellitus (T1D and T2D, respectively), and is one of the most common causes of end-stage renal disease (ESRD) worldwide [1, 2]. It is a progressive condition that gradually impairs kidney function. Clinically, DN is diagnosed in people with persistent elevation in urinary albumin (> 300 g/24 h) and a progressive decline in glomerular filtration rate (GFR). It has been shown to be associated with an elevated risk of cardiovascular morbidity and mortality [1]. Due to its progressive nature, early detection of DN is critical to have better treatment outcomes. Therefore, the need for identifying additional diagnostic and prognostic markers is essential [3].

The current diagnostic markers of DN are not reflective of direct renal injury and are insensitive to small changes in renal function [4]. For instance, the level of microalbuminuria shows considerable daily variations, which are caused by other conditions such as exercise, diet, infection, and high blood pressure [5]. Therefore, measuring microalbuminuria is an imprecise predictor for the progression or regression of DN [4]. Hence, the use of microalbuminuria in the early detection of diabetic renal lesions is questionable [6]. Serum creatinine (sCr)) is also widely used for diagnosing and monitoring various renal conditions including DN. However, its level is influenced by age, gender, muscle mass, and hydration levels and the changes in its serum level may be observed only after a substantial amount of renal function is lost. Hence the reliability of using SCr as a diagnostic tool is also questionable [7–10]. This explains the necessity to identify biomarkers that are more specific for early diagnosis and progression of kidney related diseases.

First identified in 1993 and purified from neutrophil granules [11], the neutrophil gelatinase-associated lipocalin (NGAL) is a promising marker for acute kidney injury (AKI) and kidney disease [12]. NGAL is a 25 kDa protein that belongs to the lipocalin superfamily and is encoded by the Lipocalin-2 (LCN2) gene. Similar to other members of the lipocalin family, NGAL acts as a transporter for small hydrophobic molecules and is involved in many physiological processes, such as modulating inflammation, innate immune response, and maintaining metabolic homeostasis [13, 14]. Several studies reported a significant increase in the level of NGAL in serum and urine associated with various renal pathological conditions. Therefore, NGAL has emerged as a potential biomarker for kidney dysfunction and AKI [15]. In patients with nephropathy and T1D or T2D, NGAL levels were significantly elevated in serum and urine, and it inversely correlated with the estimated glomerular filtration rate (eGFR) [16, 17]. However, these patients had normal albuminuria, implicating the potential role of NGAL as a diagnostic biomarker for DN [17].

The insulin-like growth factor (IGF) system is known to be involved in kidney growth, structure, and function. Several studies investigated the role of insulin-like growth factor-binding proteins (IGFBPs) that act as carriers and modulators for IGF-1 in the renal system. IGFBP-1, − 2, − 4, and − 5 are predominantly produced and expressed in the glomerulus, whereas IGFBP-3 and -6 are chiefly expressed in the kidney cortex [18]. Previously, it was reported that patients with DN, showed a significant increase in IGFBP-1 serum level, while the level of IGFBP-3 was low compared with control subjects [19, 20]. This dysregulation sheds light on the potential of using IGFBPs as promising diagnostic biomarkers for DN.

The aim of the current study was to analyze the circulating levels of NGAL and various IGFBPs in T2D patients with or without nephropathy and compare them to non-diabetic individuals. Furthermore, the possible association between NGAL and IGFBPs in relation to DN was investigated to identify if they could serve as sensitive biomarkers for DN.

## Materials and methods

### Study population

A total of 159 Kuwaiti participants were enrolled in this study. The study involved three main groups: 67 participants with diabetic nephropathy (DN), 50 with Type 2 diabetes (T2D) and no kidney disease and 42 non-diabetic individuals matched for age and body mass index (BMI). Clinical diagnosis of T2D was based on having persistent hyperglycemia (fasting glucose level > 7 mmol/L and 2-h FBG > 11 mmol/L) with normal kidney function. People with DN were clinically diagnosed by a nephrologist according to the American Diabetes Association criteria [21]. These patients showed pronounced T2D and persistent elevation in ACR > 30 mg/g and/or sustained reduction in estimated glomerular filtration rate (eGFR; < 60 ml/min per 1.73 m2). Patients’ exclusion criteria included: 1) Non-diabetic kidney disease, 2) Chronic liver disease, 3) Heart failure, 4) Current/recent infection, 5) Acute/chronic inflammatory disease, 6) Allergic condition, 7) Autoimmune disease, 8) Malignancy. Non- diabetic participants, who underwent routine medical check-ups at the Amiri Hospital, Kuwait, and had no history of T2D or DN and were not diagnosed with any other medical conditions were recruited as controls. This study was approved by the Ethical Review Committee of Dasman Diabetes Institute and were in accordance with the guidelines of the Declaration of Helsinki. All participants were recruited at Dasman Diabetes Institute (Dasman, Kuwait) and gave written informed consents before enrollment in the study.

### Sample collection and biochemical measurements

Blood and urine samples were obtained from participants under fasting conditions (8–12 h). Blood samples were collected in vacutainers containing Ethylenediaminetetraacetic acid (EDTA) and centrifuged at 400×g for 10 min. The Plasma was separated, aliquoted and stored at − 80 °C for further analysis. First void urine samples were collected in 120 mL urine collection tubes in the morning. The blood pressure readings presented are the average of three consecutive measurements, with a 10-min rest period between each reading (digital sphygmomanometer, Omron HEM-907XL Digital). Fasting blood glucose (FBG), triglyceride (TG), total cholesterol (TC), low-density lipoprotein (LDL), and high-density lipoprotein (HDL) were quantified using the chemistry analyzer (Siemens Dimension RXL; Diamond Diagnostics, Holliston, MA). Concentrations of urinary albumin “spot” and creatinine in addition to urinary albumin-to-creatinine ratio were measured using an automated analyzer (CLINITEK Novus Automated Urine Chemistry Analyzer; Siemens Healthineers, Erlangen, Germany). Fully automated renal function tests (RFTs) were performed using a VITROS 250 automatic analyzer. The estimated glomerular filtration rate (eGFR) was calculated using the modification of diet in renal disease study (MDRD) equation [22].

### Measurement of NGAL and IGFBPs

Stored plasma samples were thawed and centrifuged at 10,000×g for 5 min at 4 °C to remove any debris. The level of NGAL, IGFBP-1, − 3, and − 4 were measured using customized Human Magnetic Luminex Assays (LXSAHM-11 and LXSAHM-13; R&D Systems Europe, Ltd., Abingdon, UK). As per the manufacturer’s assurance, the Luminex assay kits are accurate and consistent. They perform rigorous assay validation for specificity, precision, stability, linearity and recovery to ensure high quality results. The plasma samples were diluted 50× with sample diluent for the multiplex assay to detect NGAL. To detect the different IGFBPs, plasma was diluted 2x with sample diluent. The assays were performed following manufacturer’s instructions. The results were obtained by running the assays on the Bio-Plex 200 system (Bio-Rad, CA, USA), and the Bio-Plex manager software was used to quantify the concentration of each analyte through the generated standard curve. It has been reported that the reference range for NGAL in healthy male population aged 41–60 yrs. is 103.95 ± 13.39 to 123.7 ± 15.94 μg/L. While, the reference range in healthy females is 158.37 ± 20.44 to 163.62 ± 21.12 μg/L [23]. The detection limits for IGFBP4 are 70–51,300 pg/mL and for NGAL the detection limits are 38–28,130 pg/mL. For both assays, results were calculated using the 5 PL non-linear setting in the Bioplex software with a confidence range between 75 and 110%. The standard values in the assays show 95–100% confidence range. The inter and intra-assay CVs for IGFBP4 was < 3% and the inter and intra-assay CV for NGAL was ≤5%.

### Statistical analysis

One-way analysis of variance (ANOVA) was used to compare the three study groups, namely: non-diabetic (healthy controls), T2D and DN, Bonferroni post hoc test was used for multiple comparisons to determine pair-wise statistical significance. All data are presented as mean ± standard deviation (SD). Spearman’s correlation analysis was used to evaluate the univariate association between NGAL and other biomarkers. The analysis was also used to evaluate the correlation between IGFBPs and renal function. Statistical assessment was considered significant with *p* <  0.05. Data was statistically analyzed with IBM Corp. (2017) IBM SPSS Statistics for Windows, version 25.0 (Armonk, NY: IBM Corp.). Received operating curve (ROC) analysis was performed to study the utility of NGAL and IGFBP-4 as markers for people with DN. To obtain statistical measures of the NGAL and IGFBP-4 the scales were categorized based on the elevated serum creatine or ACR to assess the AUC analysis of ROC curves. We calculated 95% CIs from the ROC analysis results based on cut-off points established. The ROC analysis results were interpreted as follows: AUC < 0.70, low diagnostic accuracy; AUC in the range of 0.70–0.90, moderate diagnostic accuracy; and AUC ≥0.90, high diagnostic accuracy.

## Results

The physical and biochemical characteristics of the study population were analyzed using one-way ANOVA and the data are presented in Table 1 as the findings of study population. The results showed no significant difference in systolic and diastolic blood pressure. There was a significant difference in FBG level between people with T2D, DN and non-diabetic control (Table 1, *p* <  0.001), with the highest values presented in people with DN. Levels of glycated hemoglobin (HbA1c) were higher in people with T2D and DN compared to the non-diabetic group (Table 1, *p* <  0.05). People with T2D and DN showed a significant increase in both TG and vLDL (*p* <  0.001), while a significant reduction in the levels of TC, LDL and HDL (Table 1, *p* <  0.01).

**Table 1:** Physical and Biochemical characteristics of the study population

Marker	Non-diabetic group (± SEM) ***N*** = 42	T2D group (±SEM) (***n*** = 50)	DN group (±SEM) (***n*** = 67)	ANOVA (***P*** value)	Multiple comparisons with post hoc Bonferroni (***P*** value)		
					T2D vs. DN	T2D vs. Healthy	DN vs. Healthy
**Age in years**	57.74 ± 1.32	58.96 ± 1.02	59.09 ± 1.38	0.015	1.000	0.104	0.014
**Gender (M % / F%)**	43 / 57	44 / 56	41 / 59				
**Body Mass Index**	33.20 ± 0.69	33.94 ± 0.88	34.23 ± 0.85	0.001	1.000	0.006	0.001
**SBP (mmHg)**	122.50 ± 2.25	132.98 ± 3.88	132.03 ± 3.41	0.087	1.000	0.139	0.162
**DBP (mmHg)**	73.76 ± 1.52	69.72 ± 2.26	68.78 ± 1.98	0.21	1.000	0.574	0.261
**Fasting Glucose (mmol/l)**	5.52 ± 0.12	8.27 ± 0.36	9.61 ± 0.48	< 0.001	0.050	0.0001	0.0001
**HbA1C (%)**	5.66 ± 0.09	9.53 ± 1.73	8.09 ± 0.22	0.031	0.816	0.027	0.236
**T chol (mmol/l)**	4.79 ± 0.15	4.15 ± 0.0.13	4.02 ± 0.0.12	< 0.001	1.000	0.006	0.0001
**TG (mmol/l)**	1.07 ± 0.08	1.41 ± 0.16	1.77 ± 0.11	< 0.001	0.102	0.202	0.001
**HDL (mmol/l)**	1.44 ± 0.06	1.25 ± 0.0.05	1.13 ± 0.0.03	< 0.001	0.207	0.026	0.0001
**LDL (mmol/l)**	3.57 ± 0.73	2.28 ± 0.11	2.1 ± 0.10	0.01	1.000	0.050	0.011
**VLDL (mmol/l)**	0.43 ± 0.03	0.56 ± 0.06	0.71 ± 0.04	< 0.001	0.105	0.196	0.0001
**Serum Creatinine (μmol/l)**	75.69 ± 2.89	79.42 ± 3.54	118.36 ± 6.57	< 0.001	0.0001	1.000	0.0001
**BUN (mmol/l)**	5.02 ± 0.20	5.10 ± 0.29	7.53 ± 0.52	< 0.001	0.001	1.000	0.0001
**eGFR MDRD (mL/min /1.73 m**^**2**^**)**	81.07 ± 2.14	79.22 ± 3.19	59.7 ± 3.00	< 0.001	0.0001	1.000	0.0001
**Albumin (g/l)**	40.5 ± 0.52	37.94 ± 0.50	37.28 ± 0.42	< 0.001	0.927	0.002	0.0001
**Urine Creatinine (mg/l)**	14.75 ± 1.22	11.94 ± 0.86	9.08 ± 0.77	0.049	0.071	0.140	0.001
**Microalbumin (mg/l)**	14.82 ± 2.0	14.35 ± 1.61	490.72 ± 186.62	< 0.001	0.003	1.000	0.005
**ACR (mg/g)**	9.77 ± 1.2	11.32 ± 1.07	953.48 ± 327.78	0.004	0.013	1.000	0.020
**NGAL (mg/l)**	52.47 ± 2.9	57.22 ± 8.7	92.76 ± 7.5	< 0.001	0.002	1.000	0.001
**IGFBP-1 (mg/l)**	19.01 ± 2.2	31.38 ± 4.7	34.85 ± 3.3	0.0091	1.000	0.080	0.008
**IGFBP-3 (mg/l)**	66.01 ± 32.4	64.99 ± 27.8	69.65 ± 24.8	0.425	0.661	1.000	1.000
**IGFBP-4 (ng/ml)**	273.06 ± 27.8	374.56 ± 86.8	795.61 ± 130.7	< 0.001	0.013	1.000	0.003
**Medications**							
**• ARBs (%)**	None	85.3	76.2				
**• ACEi (%)**		14.7	23.8				

Data are presented as mean ± SEM. One-way ANOVA was used to compare various clinical and biochemical parameters (*n* = 156). *SBP* systolic blood pressure, *DBP* diastolic blood pressure, *HbA1c* hemoglobin A1c, *Tchol* total cholesterol, *TG* triglycerides, *HDL* high-density lipoprotein, *LDL* low-density lipoprotein, *VLDL* very low density lipoprotein, *BUN* blood urea nitrogen, *eGFR* glomerular filtration rate, *ACR* urine albumin to creatinine ratio, Bonferroni post-hoc analysis was performed to compare the groups. Statistical significance is presented when *p*-value < 0.05

### Differential levels of NGAL and IGFBPs in plasma

**Fig. 1:**
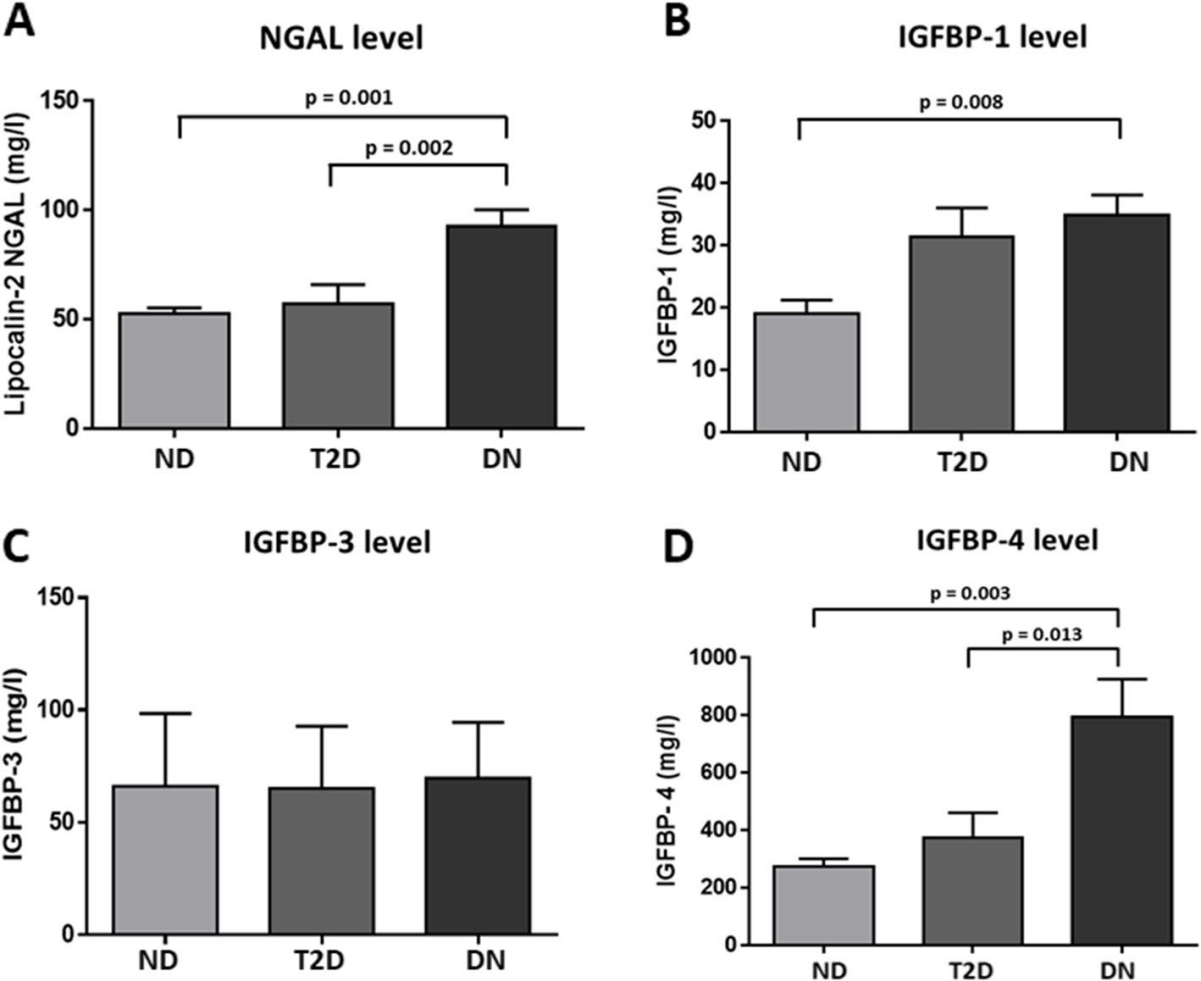
Circulatory levels of NGAL, IGFBP-1, IGFBP-3, and IGFBP-4the 3 study groups: non-diabetic (*n* = 42), T2D (*n* = 50) and DN (*n* = 67). **A** Circulating level of NGAL **B** Circulating level of IGFBP-1 (C) Circulating level of IGFBP-3 (D) Circulating level of IGFBP-4, statistical significance is presented when *p*-value < 0.05

In this study, the level of NGAL in plasma was investigated. The data showed a significant increase in plasma level of NGAL in people with DN (92.76 ± 7.5 mg/L, Table 1) compared to people with T2D (57.22 ± 8.7 mg/L; *p* = 0.002, Table 1) and non-diabetic individuals (52.47 ± 2.9 mg/L; *p* = 0.001, Table 1) (Fig. 1A). The levels of different IGFBP proteins were also analyzed. The results showed a significant increase in the level of IGFBP-4 in people with DN (795.61 ± 130 ng/ml) compared to both, people with T2D (374.56 ± 86.8 ng/ml, *p* = 0.013) and non-diabetic group (273.06 ± 27.8 ng/ml, *p* = 0.003) (Table 1, Fig. 1D). On the other hand, a significant increase in the level of IGFBP-1was seen among the DN (34.85 ± 3.3 mg/l) when compared to people from the non-diabetic group (19.01 ± 2.2, *p* = 0.008). However, no significant difference was observed between DN and T2D groups (31.38 ± 4.7 mg/l) (Table 1, Fig. 1B) IGFBP-3 expression levels showed no significant differences between the three groups (Table 1, Fig. 1C).

### Correlation analysis of NGAL with kidney function parameters

**Fig. 2:**
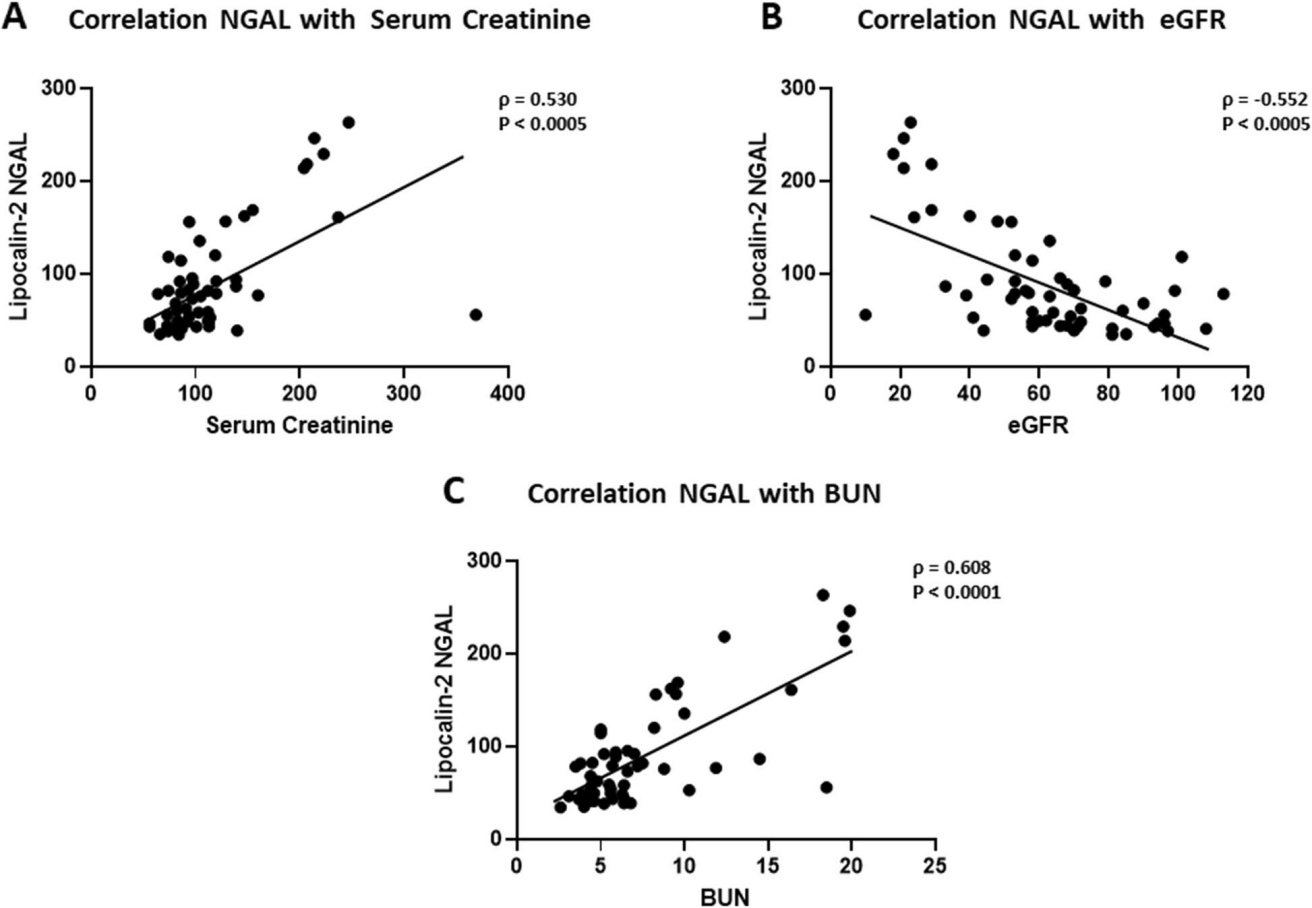
Correlation analysis between NGAL and kidney function parameters in DN group. **A** Spearman’s rank correlation between serum creatinine (SCr) and NGAL (**B**) Spearman’s rank correlation between eGFR and NGAL (**C**) Spearman’s rank correlation between BUN and NGAL. (Spearman’s correlation coefficient ρ and p-value are presented. The Association is considered statistically significant when *p*-value < 0.05)

**Fig. 3:**
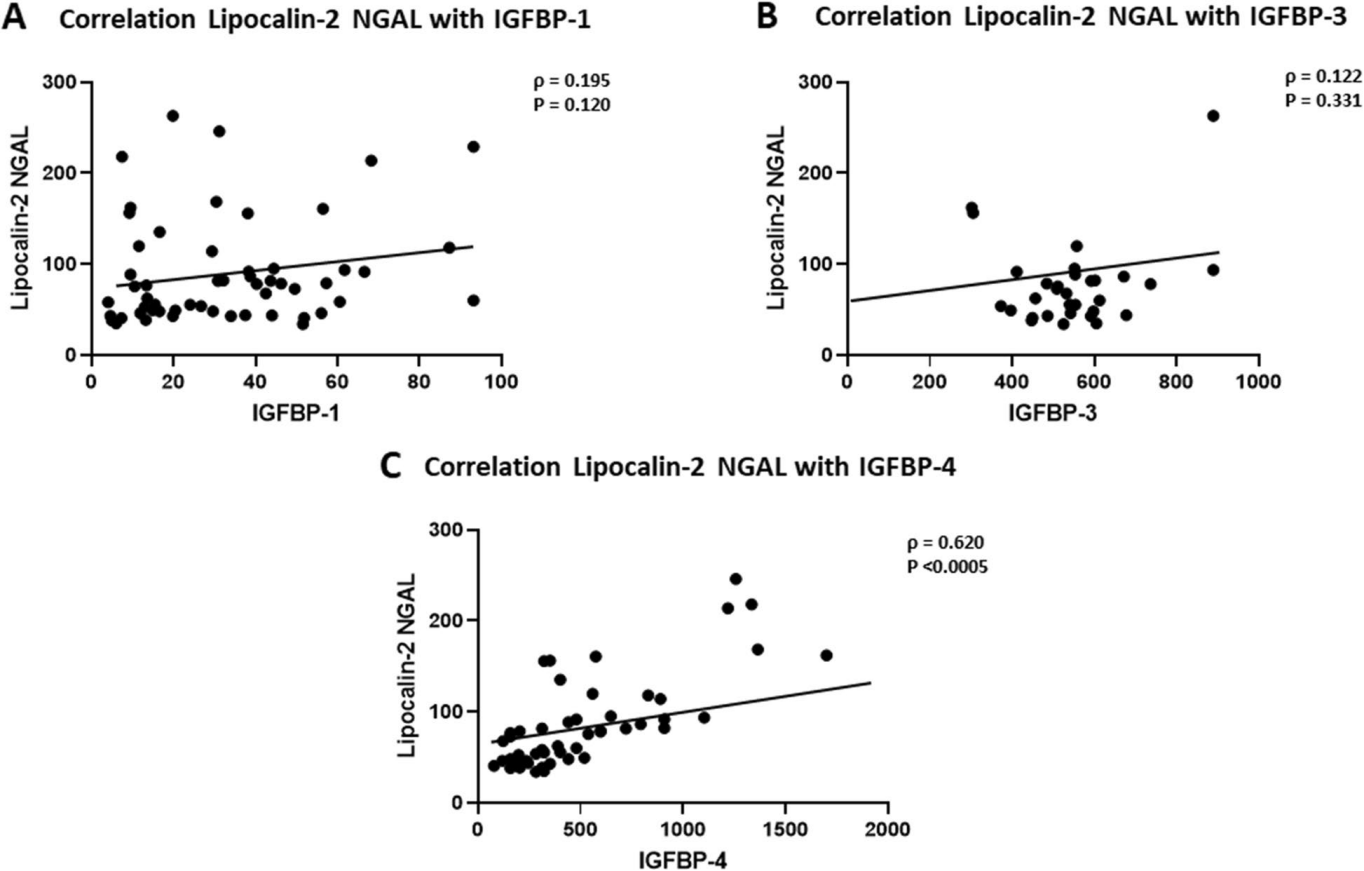
Correlation analysis between NGAL and IGFBPs in people with DN. **A** Spearman’s rank correlation coefficient between IGFBP-1 and NGAL (**B**) Spearman’s rank correlation coefficient between IGFBP-3 and NGAL (**C**) Spearman’s rank correlation coefficient between IGFBP-4 and NGAL (Spearman’s correlation coefficient ρ and p-value are presented. The Association is considered statistically significant when *p*-value < 0.05)

Spearman’s correlation analysis showed a significant association between NGAL and various markers of kidney function (Table 2) within the DN group. The data showed that NGAL was positively correlated with SCr (*ρ* = 0.53, *p* <  0.001, Fig. 2A), while it correlated negatively with eGFR (*ρ* = − 0.552, *p* <  0.001, Fig. 2B). A positive correlation was also observed between NGAL and the blood urea nitrogen (BUN) (*ρ* = 0.608, *p* <  0.001, Fig. 2C). Interestingly, the analysis revealed a significant correlation between NGAL and IGFBP-4 in people with DN (*ρ* = 0.62, *p* <  0.001, Fig. 3C). No significant associations between NGAL and IGFBP1/3 (Fig. 3A and B respectively). In a similar manner, NGAL showed a significant association with parameters of kidney function in people with T2D (Table 2). The correlation was positive with SCr (*ρ* = 0.63, *p* <  0.001), and BUN (*ρ* = 0.577, *p* <  0.001) but negative with eGFR (*ρ* = − 0.707, *p* <  0.001). There was no significant correlation between NGAL and IGFBP-4 in people with T2D (Table 2). Furthermore, no significant association between NGAL and urinary proteins was found.

**Table 2:** Spearman’s rank correlation between NGAL with Serum Creatinine, BUN, eGFR and IGFBPs

Markers	Non-diabetic group		T2D group		DN group	
	ρ	*P* value	ρ	*P* value	ρ	*P* value
**Serum Creatinine**	0.381	< 0.05	0.631	< 0.001	0.530	< 0.001
**BUN**	0.195	0.221	0.577	< 0.001	0.608	< 0.001
**eGFR**	- 0.430	< 0.001	- 0.707	< 0.001	- 0.552	< 0.001
**IGFBP-1**	0.010	0.950	0.167	0.256	0.195	0.120
**IGFBP-3**	0.005	0.976	- 0.113	0.450	0.122	0.331
**IGFBP-4**	0.019	0.906	0.252	0.085	0.620	< 0.001

*IGFBP* Insulin-like growth factor binding protein −1, 3, 4, *BUN* blood urea nitrogen, *eGFR* glomerular filtration rate, Spearman’s correlation coefficient ρ and *p*-value are presented. The Association is considered statistically significant when *p*-value < 0.05

### Correlation analysis of IGFBPs with kidney function parameters

Spearman’s rank correlation analysis was performed to study the correlation between the IGFBPs and the various indicators of renal activity. A significant correlation was observed between IGFBP-4 and the indicators of renal activity within the DN group. This was presented through a significant positive correlation with serum creatinine (*ρ* = 0.39, *p* <  0.001), BUN (*ρ* = 0.32, *p* <  0.05), and a negative correlation with eGFR (*ρ* = − 0.44, *p* <  0.001). In the case of IGFBP-1, the data showed a significant negative correlation with both sCr (*ρ* = − 0.249, *p* <  0.05) and BUN (*ρ* = − 0.371, *p* <  0.001) in people with DN, whereas eGFR showed no association with IGFBP-1 in the same group (Table 3). No association was found between IGFBP-3 and the parameters reflecting kidney function in people with DN (Table 3). Furthermore, the relationship between IGFBP4 and urinary protein excretion (urine creatinine, microalbumin and ACR) was assessed. IGFBP4 was found to associate significantly only with urine Creatinine in the DN group (*ρ* = − 0.289, *p* = 0.021, Supplementary Table 1).

**Table 3:** Spearman’s rank correlation between IGFPBs and renal markers in the non-diabetic, T2D and DN groups

Study group	Markers	IGFBP-1		IGFBP-3		IGFBP-4	
		ρ	*P* value	ρ	*P* value	ρ	*P* value
**Non-diabetic group**	Serum Creatinine	- 0.039	0.807	- 0.176	0.270	0.302	0.052
	BUN	0.047	0.769	0.212	0.183	- 0.038	0.809
	eGFR	- 0.010	0.948	0.177	0.267	- 0.306	0.049
**T2D group**	Serum Creatinine	- 0.006	0.966	- 0.034	0.814	0.269	0.059
	BUN	0.160	0.267	0.002	0.991	- 0.013	0.927
	eGFR	- 0.141	0.334	- 0.036	0.794	−0.271	0.059
**DN group**	Serum Creatinine	- 0.249	< 0.05	0.178	0.150	0.397	< 0.001
	BUN	- 0.371	< 0.001	0.194	0.116	0.327	< 0.05
	eGFR	0.171	0.166	- 0.206	0.095	- 0.448	< 0.001

*IGFBP* Insulin-like growth factor binding protein −1,3,4, *BUN* blood urea nitrogen, *eGFR* glomerular filtration rate, Spearman’s correlation coefficient ρ and *p*-value are presented. The Association is considered statistically significant when *p*-value < 0.05

### ROC analyses for NGAL and IGFBP4

**Fig. 4:**
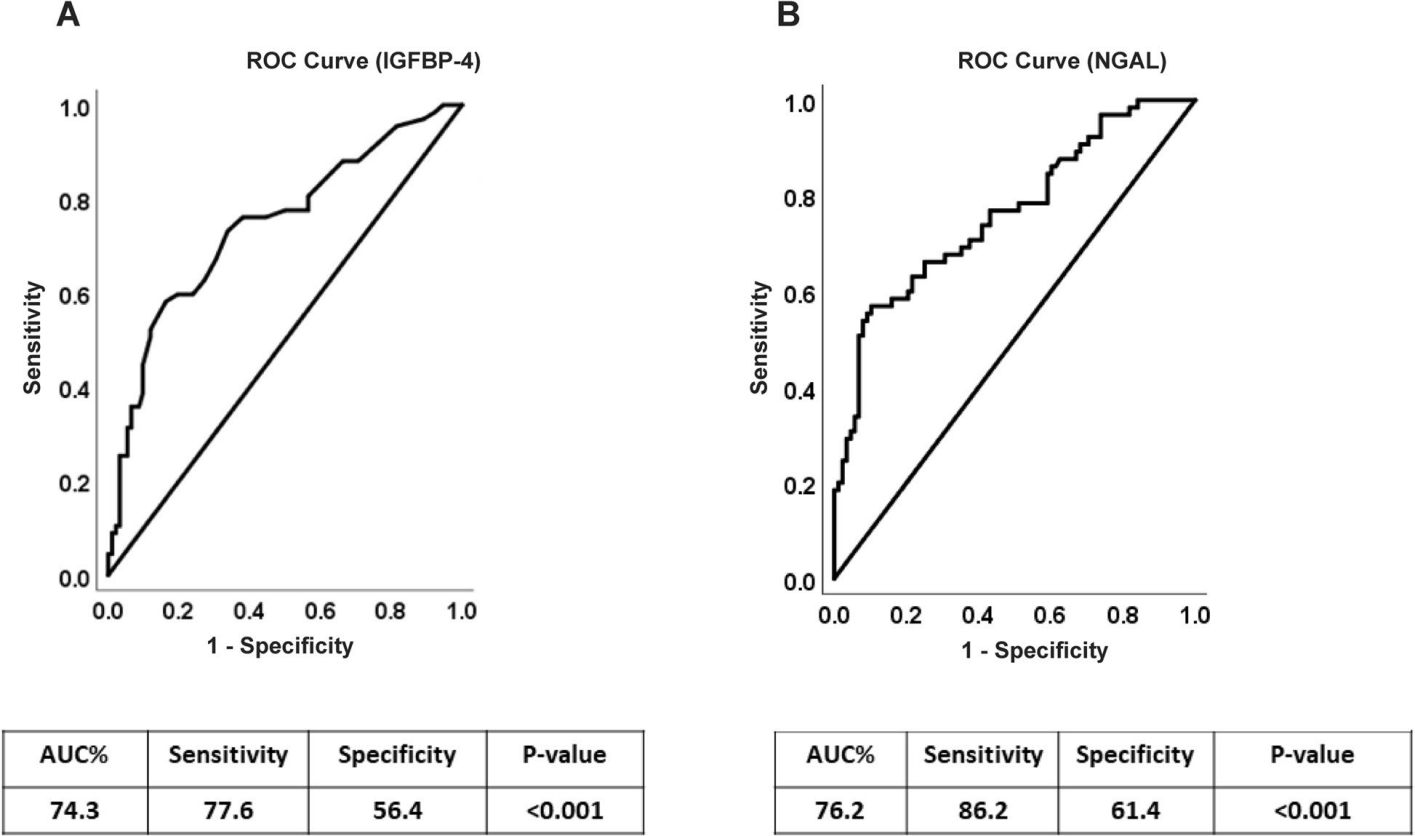
Receiver Operating Characteristic curves for IGFBP-4 (**A**) and the NGAL (**B**) based on the diagnosis of DN

Receiver Operating Characteristic (ROC) analysis was performed to determine the probable use of NGAL and IGFBP4 markers to differentiate between diabetic patients with or without diabetic nephropathy. NGAL and IGFBP4 markers show moderate diagnostic accuracy considering the ROC analysis results and the fact that AUC for both markers were 76.2 and 74.3 respectively (Fig. 4A and B). Moreover, ROC curve analyses showed significant predictive power of the ACR and SCr for NGAL and IGFBP4 markers, with SCr exhibiting high accuracy (supplementary Fig. 1A and B) and ACR exhibiting low accuracy (Supplementary Fig. 1C and D). The AUCs, *p*-values, cut-off points optimized for sensitivity and specificity, 95% CIs, sensitivities, and specificities obtained for SCr and ACR are reported in Supplementary Table 2. The ROC analysis results were interpreted as follows: AUC < 0.70, low diagnostic accuracy; AUC in the range of 0.70–0.90, moderate diagnostic accuracy; and AUC ≥0.90, high diagnostic accuracy. We also created ROC curve for serum creatinine as one of the conventional circulatory markers to assess the diagnostic utilities of the NGAL and IGFBP4 circulatory molecules (Supplementary Fig. 2).

### Assessing the levels of NGAL and IGFBP4 in normal creatinine group

The study groups namely non-diabetic, T2D and DN were further categorized based on creatinine levels into 2 sub-categories as normal (Male <=119.3; Female <=91.9) and high (Male > 119.3; Female > 91.9). Plasma levels for both NGAL and IGFBP4 seem to be increased in both subgroups. However, their increased in levels were more pronounced in the high creatinine subgroup (Supplementary Table 3). Moreover, and within the normal creatinine subgroup, it was evident the increased of both NGAL and IGFBP4 levels in the DN group compared to both DM and non-diabetic ones supporting the utility of both markers for early diagnosis of DN.

## Discussion

During the past decade, the prevalence of diabetes has escalated, affecting 415 million people worldwide. DN is a common complication associated with T1D and T2D, and it is regarded as the leading cause of chronic kidney disease (CKD) worldwide [24]. In addition to the progress in the available management and treatment options, finding biomarkers with higher sensitivity would greatly reduce the prevalence of DN and the incidence of diabetes related ESRD. Currently, microalbuminuria and serum creatinine are the diagnostic markers for DN, however they exhibit some limitations that affect their diagnostic efficiency [5, 8–10]. Indicating the need for identifying additional diagnostic and prognostic markers to achieve better management of DN. In the present study a significant increase in the circulatory level of NGAL was observed among individuals with DN. This increase was significantly associated with elevated level of IGFPB-4 and renal indicators in people with DN. Only people with DN showed significant increase in NGAL and IGFPB-4 levels, suggesting them as potential diagnostic and prognostic markers for DN.

The pathophysiology of DN has been attributed to multifactorial interactions between metabolic and hemodynamic factors, including glucose-dependent pathways and the renin–angiotensin system [25]. Such molecular abnormalities may damage the glomerulus and renal tubulointerstitium, which contribute to elevated levels of NGAL and other renal markers, including SCr and BUN. Compared to the routinely used renal markers, NGAL is a sensitive biomarker for AKI that increases significantly in the blood and urine within 2 h of injury [26]. As such, elevated NGAL levels marks the occurrence of AKI [27]. Increased levels of plasma NGAL showed a predictive power for CKD progression and was reflective of renal disease severity [28, 29]. In this study, a rise in NGAL and IGFBP-4 levels was evident in people with DN even in those demonstrating normal creatinine levels, the increase in circulating NGAL in people with DN reflected its importance as an indicator of DN. This was accentuated by the significant positive correlation with IGFBP-4 in people with DN, a marker that has been linked with DN [30, 31]. Conventional renal markers like Cr and microalbumin are important for the diagnosis and staging of kidney disease. However, a significant change in conventional markers is usually detected after the occurrence of a substantial glomerular damage. Although, NGAL is a rapid marker for the detection of an acute renal injury, changes in NGAL levels were not reflective of a kidney functional deficit [15]. Nonetheless, others found that changes in NGAL levels are predictive of CKD progression and severity [28].

Knowing that the IGF system is involved in CKD pathology [18], and IGF-1 is one of the main factors involved in the development of DN, the aim of this study was to investigate the involvement of other IGFBPs and their contribution to the pathology of a diabetic kidney. IGFBP-4, which is typically expressed in the kidneys, has been suggested as a marker for autoimmune diseases, including chronic lupus nephritis [32]. Wu et al. showed a positive correlation between circulating IGFBP-4 and SCr levels and an inverse correlation with eGFR in patients with lupus nephritis. In the current study cohort, people with DN showed a significant elevation in levels of IGFBP-4 compared with the control or people with T2D [31]. The rise in IGFBP-4 levels demonstrated a significant association with renal markers including, SCr, BUN and eGFR. This came in agreement with previous reports and it supports the potential involvement of IGFBP-4 in diabetic kidney disease [30].

Collectively, this study highlights significant increase in the levels of two biomarkers, NGAL and IGFBP-4, in people with DN, implicating their involvement in renal pathology. This elevation was not prominent in people with T2D and was not present in the non-diabetic group. The potential link of these proteins in DN was accentuated by the significant positive correlation between IGFBP-4 and NGAL in people with DN, which could imply their potential use as biomarkers aiding in the early detection of DN. Our results indicated that NGAL and IGFBP4 plasma levels are high in patients with diabetic nephropathy. Whether these markers are superior to the ratio of albumin to creatinine in providing earlier diagnosis for diabetic nephropathy require further analysis including annual follow-ups and monitoring before diabetic nephropathy develops. The current study is limited by its cross-sectional study design and the small sample size of the cohorts. Thus, future studies are required to elucidate the mechanism through which NGAL and IGFBP-4 contribute towards the progression and manifestation of DN and asses their possible inflammatory role. One of the main limitations of this study is that it does not include CKD patients who are not diabetic. Inclusion of this category of patients would allow better understanding on whether these markers are DN specific or if they can also detect other kidney impairments.

## Conclusion

This report highlights the dysregulation and positive association between two proteins, NGAL and IGFBP-4 in circulation and their potential as biomarkers for DN. In the field of nephrology, DN continues to be a major condition that is best managed by early detection to prevent the occurrence of irreversible kidney damage. The data from this study presented circulating markers as potential diagnostic tools for DN. Future studies are required to investigate the specific role of these markers and their feasibility as early diagnostic tools for kidney- related diseases.

## Supplementary Information

**Additional file 1.**

**Additional file 2.**

**Additional file 3.**

## Data Availability

The datasets generated during and/or analyzed during the current study are not publicly available but are available from the corresponding author on reasonable request.
