# Supplementary material for: Association of significantly elevated plasma levels of NGAL and IGFBP4 in patients with diabetic nephropathy
Source: BMC Nephrol. 2022 Feb 11;23:64. doi: 10.1186/s12882-022-02692-z (PMC8840773; doi:10.1186/s12882-022-02692-z)
Supplement: Supplementary file 1 — Additional file 1. [file 12882_2022_2692_MOESM1_ESM.docx]

**Supplementary Material Folder**

**Supplementary tables**

**Supplementary table 1.** Spearman's rank correlation between IGFPB4 and NGAL and the urinary proteins in the non-diabetic, T2D and DN groups.

| **Study group** | **Urine Markers** | **Non-diabetic group** | | **T2D Group** | | **DN Group** | |
| --- | --- | --- | --- | --- | --- | --- | --- |
|  |  | ρ | *P value* | ρ | *P value* | ρ | *P value* |
| **NGAL** | Urine Creatine | 0.042 | 0.794 | -0.032 | 0.836 | -0.109 | 0.399 |
|  | Microalbumin | -0.054 | 0.739 | 0.207 | 0.172 | 0.072 | 0.578 |
|  | ACR | -0.037 | 0.816 | 0.241 | 0.110 | 0.092 | 0.475 |
| **IGFBP-4** | Urine Creatine | 0.036 | 0.821 | 0.058 | 0.696 | -0.289 | 0.021 |
|  | Microalbumin | -0.005 | 0.974 | 0.209 | 0.159 | -0.005 | 0.967 |
|  | ACR | -0.017 | 0.917 | 0.160 | 0.282 | 0.029 | 0.819 |

ACR (urine albumin to creatinine ratio); NGAL (neutrophil gelatinase-associated lipocalin) ; IGFBP-4 (Insulin-like growth factor binding protein -4); Spearman’s correlation coefficient ρ and p-value are presented. The Association is considered statistically significant when p-value <0.05

**Supplementary table 2.** ROC Analysis optimized for Serum Creatinine and ACR

|  | **Group** | **AUC** | **P value** | **Cut-off** | **95% CI** | **Sensitivity** | **Specificity** |
| --- | --- | --- | --- | --- | --- | --- | --- |
| **Serum Creatinine** | NGAL | 0.79 | <0.001 | 43917.41 | 0.72 – 0.87 | 52.2 | 91.8 |
|  | IGFBP-4 | 0.74 | <0.001 | 213.50 | 0.66 - 0.83 | 51.1 | 84.0 |
| **Albumin Creatinine Ratio (ACR)** | NGAL | 0.70 | <0.001 | 43789.01 | 0.62 – 0.79 | 41.7 | 76.1 |
|  | IGFBP-4 | 0.68 | <0.001 | 240.0 | 0.59 – 0.77 | 44.0 | 75.4 |

**Supplementary table 3.** Circulatory levels of NGAL and IGFPB4 in the Non-diabetic, T2D and DN groups stratified based on the serum creatinine levels.

|  |  | **Serum Creatine-Normal** | | | **Serum Creatine-High** | | |  | |
| --- | --- | --- | --- | --- | --- | --- | --- | --- | --- |
| **GROUP** | **VARIABLES** | **N** | **Mean** | **Std. Error** | **N** | **Mean** | **Std. Error** | **P value** |  |
| Non-diabetic | NGAL | 34 | 50277.36 | 3175.78 | 7 | 63146.71 | 6591.95 | 0.112 |  |
|  | IGFBP4 | 35 | 257.06 | 30.48 | 7 | 353.06 | 63.88 | 0.208 |  |
|  |  |  |  |  |  |  |  |  |  |
| T2DM | NGAL | 37 | 47875.46 | 3352.24 | 8 | 111086.03 | 47505.05 | 0.226 |  |
|  | IGFBP4 | 38 | 298.62 | 47.27 | 9 | 749.19 | 36.55 | 0.334 |  |
|  |  |  |  |  |  |  |  |  |  |
| DN | NGAL | 24 | 64457.83 | 6304.54 | 38 | 112566.05 | 11067.99 | <0.001 |  |
|  | IGFBP4 | 24 | 566.39 | 216.87 | 40 | 959.04 | 173.28 | 0.163 |  |

NGAL (neutrophil gelatinase-associated lipocalin) ; IGFBP-4 (Insulin-like growth factor binding protein -4); the levels are considered significantly difference when p-value <0.05
