# Supplementary figures and images for: Association of significantly elevated plasma levels of NGAL and IGFBP4 in patients with diabetic nephropathy
Source: BMC Nephrol. 2022 Feb 11;23:64. doi: 10.1186/s12882-022-02692-z (PMC8840773; doi:10.1186/s12882-022-02692-z)

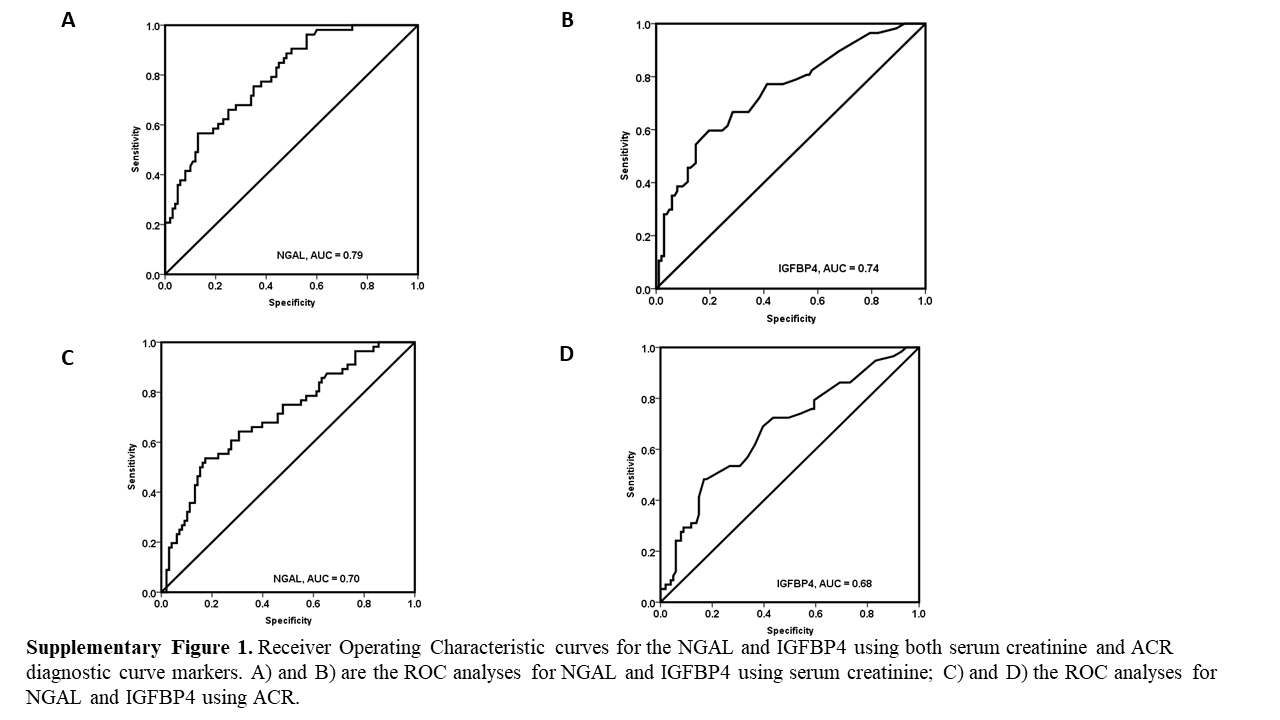

Supplement: Supplementary file 2 — Additional file 2. [file 12882_2022_2692_MOESM2_ESM.tif]

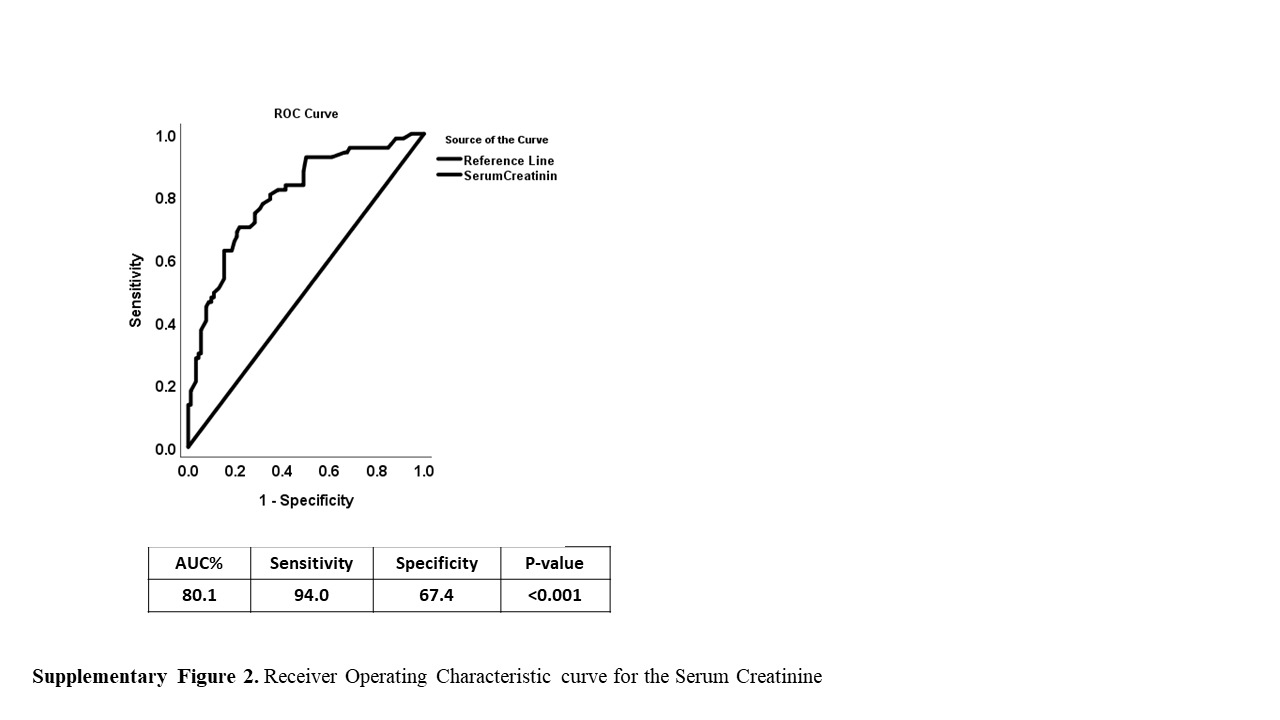

Supplement: Supplementary file 3 — Additional file 3. [file 12882_2022_2692_MOESM3_ESM.tif]
